# Allospecific Tregs Expanded After Anergization Remain Suppressive in Inflammatory Conditions but Lack Expression of Gut-homing Molecules

**DOI:** 10.1038/mt.2016.64

**Published:** 2016-05-03

**Authors:** Eleni Kotsiou, John G Gribben, Jeff K Davies

**Affiliations:** 1 Centre for Haemato-Oncology, Barts Cancer Institute, Queen Mary University London, London, UK

## Abstract

Cell therapy with antigen-specific regulatory T-cells (Treg) has great potential to selectively control unwanted immune responses after allogeneic stem-cell or solid organ transplantation and in autoimmune diseases. *Ex vivo* allostimulation with costimulatory blockade (alloanergization) of human T-cells expands populations of alloantigen-specific Treg, providing a cellular strategy to control donor T-cell alloresponses causing graft-versus-host disease after allogeneic hematopoietic stem-cell transplantation. Crucially, it is not known if Treg expanded in this way are stable in proinflammatory conditions encountered after transplantation, or if they possess capacity to migrate to key target organs. Using an *in vitro* model to functionally characterize human Treg expanded after alloanergization, we now show that these cells remain potently allosuppressive in the presence of relevant exogenous inflammatory signals. Expanded allospecific Treg retained expression of molecules conferring migratory capacity to several organs but small intestine-specific chemotaxis was markedly impaired, in keeping with the preponderance of gut graft-versus-host disease in previous clinical studies using this strategy. Importantly, impaired gut-specific chemotaxis could be partially corrected by pharmacological treatment. These findings will facilitate more effective application of this cellular approach to limit T-cell alloresponses after hematopoietic stem-cell transplantation and the wider application of the strategy to other clinical settings.

## Introduction

Regulatory T-cells (Treg) are a subset of CD4^+^ T-cells with functional ability to suppress effector T-cell immune responses. Cellular therapy with antigen-specific Treg has potential to selectively control unwanted T-cell immune responses that mediate a range of pathologies including graft-versus-host disease (GvHD) after allogeneic hematopoietic-cell transplantation (AHSCT),^1^ graft rejection after solid organ transplantation^2^ and autoimmune diseases.^3^ However, for such cellular therapy to be successful, antigen-specific Treg need to retain functional stability^4^ and capacity to migrate to anatomical sites of T-cell priming and tissue damage,^5^ particularly in proinflammatory conditions often present in these clinical settings.

Most approaches to generate and expand therapeutic antigen-specific Treg require cumbersome cell sorting procedures which can be difficult to implement at a clinical scale.^6^ An alternative strategy is the stimulation of human donor T-cells with alloantigens in the presence of antibody- or fusion protein-mediated blockade of the CD28 costimulatory signal (alloanergization).^7^ This strategy renders alloantigen-specific effector T-cells hyporesponsive while expanding allosuppressive Treg, thus generating a tolerogenic pool of T-cells without the requirement for cell purification steps. Alloanergization was initially developed for use in human leukocyte antigen (HLA)-mismatched AHSCT to limit donor T-cell alloresponses that mediate harmful GvHD without global immunosuppression,^8^ but could be applied as a simple strategy to generate antigen-specific Treg in other clinical settings.

In the two previous clinical studies of HLA-mismatched bone marrow transplantation, infusion of alloanergized donor immune cells resulted in a marked expansion of alloantigen-specific donor CD4^+^ Treg in recipient peripheral blood posttransplant.^9^ Furthermore, Treg generated in this way *in vivo* or *in vitro* possess donor-specific allosuppressive capacity without suppressing beneficial pathogen-specific immune responses.^9^ Although GvHD occurred at a lower frequency than historical controls in these clinical studies, some breakthrough acute GvHD occurred and was limited to the gastrointestinal tract.^9,10,11^ Therefore, the elucidation of mechanisms underlying the spatial or functional limitations of alloantigen-specific Treg generated after alloanergization will help improve the approach in the setting of AHSCT and may have broader significance in the application of the strategy to other clinical scenarios.

We therefore used an *in vitro* model of alloanergization to expand and scrutinize human alloantigen-specific CD4^+^ Treg. We measured the functional stability and differential tissue-specific migratory capacity of these cells after repeated exposure to alloantigens under various proinflammatory conditions to model the effects of the posttransplantation environment and to determine potential mechanisms underlying constraints on the efficacy of this form of cellular therapy.

## Results

### Expansion of CD4^+^ Treg after alloanergization is maintained in the presence of proinflammatory signals

We have shown earlier that alloanergization of human T-cells results in an expansion of the frequency of allospecific CD4^+^ Treg upon rechallenge with alloantigen.^9,12^ Proinflammatory conditions likely to be encountered *in vivo* after transplantation of alloanergized donor immune cells have recently been shown to convert induced Treg into effector T-cells.^13^ We therefore, measured the impact of proinflammatory mediators relevant to allogeneic transplantation on the numerical expansion and function of CD4^+^ Treg after alloanergization of donor T-cells.

We first determined the effect of exogenous lipopolysaccharide (LPS), which is released from the gastro-intestinal tract after AHSCT,^14^ stimulates release of tumor necrosis factor-α and interferon (IFN)-γ from monocytes and macrophages and polarizes allogeneic donor T-cells to Th1 effector responses that facilitate acute GvHD.^15^ In our experimental model, the proinflammatory effect of LPS was likely to be via this mechanism, rather than directly modulating Treg in our cultures as TLR4 was expressed by <5% of CD4^+^ Treg in starting populations or expanded after alloanergization, **Supplementary Figure S1a**.

**Figure 1:**
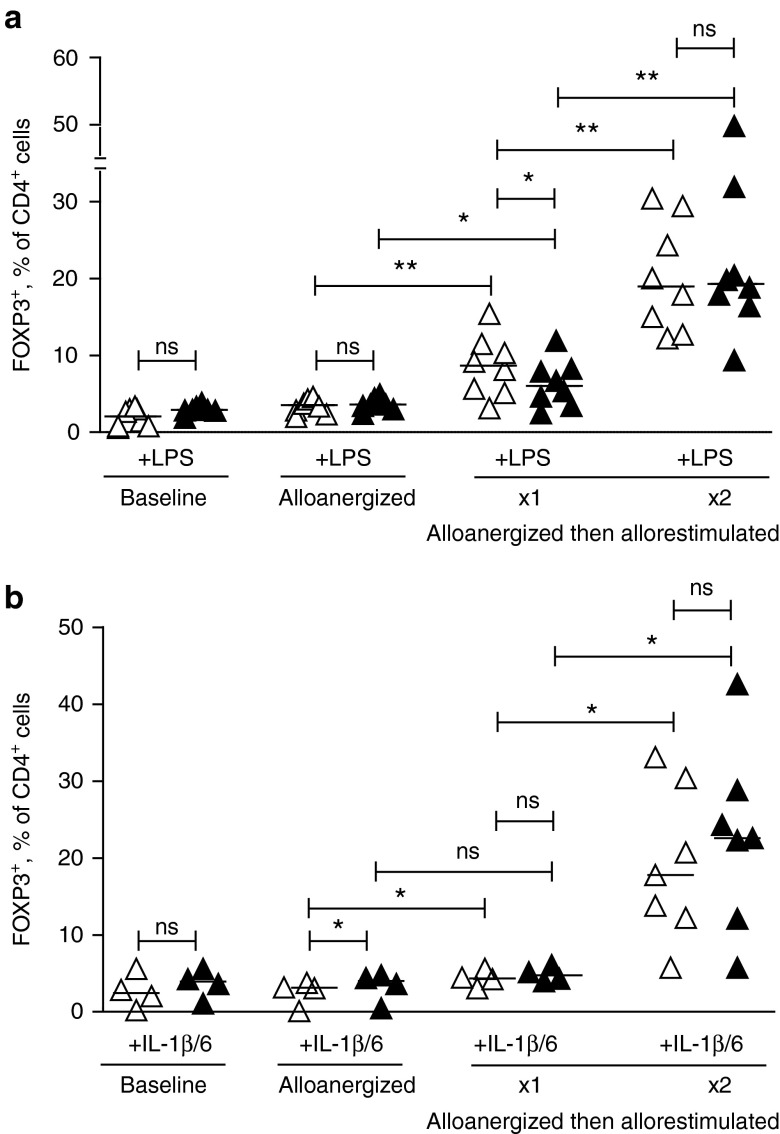
**Expansion of CD4^+^ regulatory T-cells (Treg) after alloanergization is maintained in proinflammatory conditions.** (**a**) FOXP3^+^ Treg, expressed as percentage of total CD4^+^ cells, after alloanergization and repeated exposure to alloantigen in the absence or presence of lipopolysaccharide (LPS). Graph depicts results from eight different HLA-mismatched stimulator-responder pairs. *P* values are for two-tailed student's *t*-test. Horizontal lines are medians. *P < 0.05, **P < 0.01, ns, not significant. (**b**) FOXP3^+^ Treg, expressed as percentage of total CD4^+^ cells, after alloanergization and repeated exposure to alloantigen in the absence or presence of IL-1β and Il-6. Graph depicts results from seven different HLA-mismatched stimulator-responder pairs. *P* values are for two-tailed student's *t*-test. Horizontal lines are medians. *P < 0.05, **P < 0.01, ns, not significant.

The addition of LPS did not significantly reduce the expansion of frequencies of CD4^+^ Treg (identified by expression of intracellular FOXP3 or by CD25^hi^CD127^lo^ surface phenotype) after alloanergization. Importantly, expansion of Treg was maintained when alloanergized cells were restimulated with alloantigen in the presence of LPS, modeling the postinfusional environment where alloanergized cells would encounter massive allorestimulation and local proinflammatory signals (**Figure 1a** and **Supplementary Figure S2**).

We also examined the effect of the proinflammatory cytokines IL-1β and IL-6, which are released by damaged tissues after transplant conditioning, polarize toward a Th17 immune microenvironment which has also been implicated in acute GvHD and are known to impair differentiation or induce phenotypic instability of CD4^+^ Treg.^16,17^ The expansion of CD4^+^ Treg after alloanergization and subsequent allorestimulation of donor T-cells was also maintained in the presence of exogenous IL-1β and IL-6, **Figure 1b**, despite a subpopulation of Treg expressing the receptor for IL-1 β (IL1R1, **Supplementary Figure S1b**).

### Th1-polarizing conditions do not change the phenotype of expanded Treg after alloanergization

**Figure 2:**
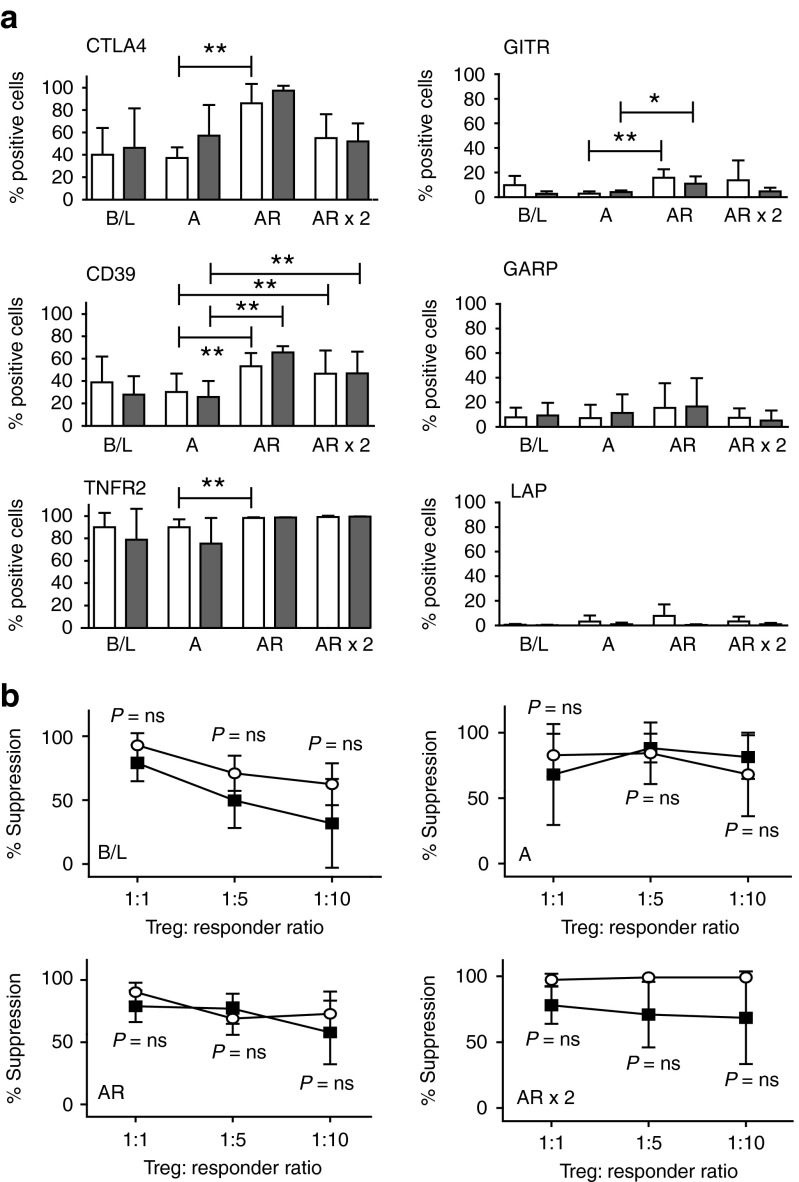
**Lipopolysaccharide (LPS) does not impair allosuppressive phenotype or function of CD4^+^ regulatory T-cells (Treg) expanded after alloanergization**. (**a**) Phenotype of expanded Treg after alloanergization and repeated exposure to alloantigen in the absence of presence of LPS. Bar charts show mean ±SD) frequencies of CTLA-4^+^, CD39^+^, TNFR2^+^, GITR^+^, GARP^+^, and LAP^+^ cells expressed as a proportion of CD4^+^ FOXP3^+^ Treg at baseline (B/L), after alloanergization (A) and after subsequent allorestimulation (AR). Data are for five to nine HLA-mismatched stimulator-responder pairs. *P < 0.05, **P < 0.01. (**b**) Allosuppressive function of CD4^+^ Treg at B/L, after alloanergization (A) and subsequent AR in the absence or presence of LPS. Mean percentage suppression (±SD) of alloproliferative responses of untreated responder cells by CD4^+^ Treg are shown. Data are for three to six HLA-mismatched stimulator-responder pairs. *P* values are for two-tailed *t*-tests, ns; nonsignificant.

After demonstrating that expansion of CD4^+^ Treg after alloanergization was not reduced by proinflammatory mediators, we next chose to phenotypically characterize expanded Treg in this setting. We measured cellular coexpression patterns of six key molecules important in CD4^+^ Treg stability and function. Allorestimulation of alloanergized cells significantly increased the frequencies of CD4^+^FOXP3^+^ Treg expressing CTLA4 (a key mediator of Treg suppression), CD39 (which hydrolyzes the important inflammatory danger signal extracellular ATP), and TNFR2 (vital for TNF-mediated stabilization of Treg function) when compared with alloanergized Treg. Frequencies of GITR^+^ Treg (known to have Th1-secreting capacity) were also significantly increased after allorestimulation of alloanergized cells although frequencies remained <20%. In contrast, GARP and LAP (which negatively control Treg expansion) were expressed at low levels in baseline Treg and expanded Treg. Importantly, the phenotype of expanded populations of Treg was maintained in the presence of exogenous LPS. Overall, the phenotypic expression patterns on expanded populations of CD4^+^ Treg after alloanergization are consistent with cell lineage stability and retention of suppressive function in proinflammatory conditions (**Figure 2a**).

### Expanded Treg retain allosuppressive function in the presence of proinflammatory conditions

After demonstrating that proinflammatory conditions did not adversely impact on numerical expansion or suppressive phenotype of Treg expanded after alloanergization we determined if allosuppressive function was also retained in this setting. CD4^+^ Treg purified immunomagnetically (based on CD25^hi^ CD127^lo^ expression, purity >95%, FOXP3^+^ >75%) were potently allosuppressive following alloanergization and subsequently allorestimulation. Importantly the addition of LPS did not significantly reduce this allosuppressive capacity of Treg expanded after alloanergization (**Figure 2b**). Furthermore, the allosuppressive capacity of expanded populations of Treg was not significantly reduced by the presence of IL-1β and IL-6 (*P* = not significant for comparison between suppressive capacity of cells with or without IL-1β and IL-6 exposure at all ratios (**Supplementary Figure S3**). This data provides evidence that therapeutic alloantigen-specific Treg generated after alloanergization are functionally stable in clinically relevant proinflammatory conditions.

### Th1-polarizing proinflammatory conditions increases expansion of allosuppressive IFN-γ^+^ Treg

Proinflammatory conditions have been shown to induce the capacity of Treg to secrete IFN-γ,^18^ which may impact on their function. Therefore, we next sought to determine if expanded populations of Treg generated after alloanergization in proinflammatory conditions were enriched for such IFN-γ secreting cells.

**Figure 3:**
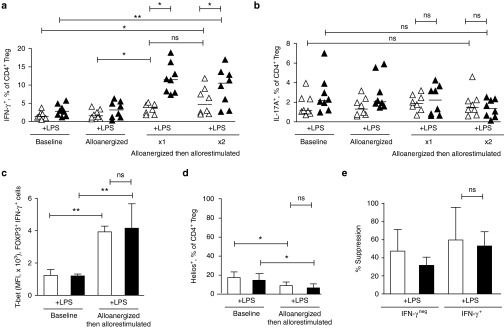
**Regulatory T-cells (Treg) expanded after alloanergization in lipopolysaccharide (LPS)-rich conditions are enriched with allosuppressive IFN-γ-secreting cells.** (**a**) Frequencies of IFN-γ^+^ cells expressed as percentage of CD4^+^ Treg at baseline, after alloanergization and after allorestimulation in the absence or presence of LPS. Horizontal lines represent median values. *P* values are for two-tailed *t*-test. Results are shown for eight HLA-mismatched stimulator-responder pairs. *P < 0.05, **P < 0.01. (**b**) Frequencies of IL-17A^+^ cells expressed as percentage of Treg at baseline, after alloanergization and after allorestimulation. Horizontal lines represent median values. *P* values are for two-tailed *t*-test. Results are shown for eight HLA-mismatched stimulator- responder pairs. ns, not significant. (**c**) IFN-γ^+^ Treg following alloanergization and allorestimulation upregulate T-bet. Bar chart summarizes mean (±SD) median fluorescence intensity (MFI) of T-bet CD4^+^FOXP3^+^IFN-γ^+^ Treg at baseline and after alloanergization and allorestimulation. Results for three independent experiments are shown. *P* values are for two-tailed *t*-test. **P < 0.01, ns, not significant. (**d**) IFN-γ^+^ Treg following alloanergization and allorestimulation are predominantly helios^neg^. Mean (±SD) frequencies of Helios^+^ cells expressed as a percentage of CD4^+^FOXP3^+^IFN-γ^+^ Treg at baseline and after alloanergization and subsequent allorestimulation. Results are for three independent experiments. *P* values are for two-tailed *t*-test. *P < 0.05, ns, not significant. (**e**) Allosuppressive capacity of IFN-γ^+^ and IFN-γ^neg^ Treg subpopulations after alloanergization and subsequent allorestimulation of healthy donor peripheral blood mononuclear cells (PBMCs) in the presence or absence of LPS. Mean percentage suppression (±SD) of alloproliferative responses of untreated responder cells are shown by purified Treg from allorestimulated alloanergized PBMCs at a ratio of 1:10 Treg: Responder cells. Results are from four independent HLA-mismatched stimulator-responder pairs. Ns, not significant.

In the absence of proinflammatory conditions, the proportion of Treg secreting IFN-γ in baseline cells increased threefold after alloanergization and allorestimulation (from a median frequency of 1.4% to a median frequency of 4.7%, *P* = 0.001) demonstrating that the process of alloanergization and allorestimulation expanded frequencies of Treg secreting IFN-γ. Importantly, the presence of LPS resulted in significantly higher proportions of IFN-γ^+^ Treg after alloanergization and allorestimulation (11% LPS versus 4.7% no LPS, *P* = 0.01) (**Figure 3a**). The increase in the proportion of IFN-γ^+^ Treg we observed was due to an increase in absolute numbers of IFN-γ^+^ Treg (rather than due to a selective decrease in IFN-γ^+^ Treg) as total viable CD4^+^ T-cell numbers were not significantly different in baseline or alloanergized allorestimulated cultures, either in the absence (8.4 ± 1.5 × 10^6^ versus 10.73 ± 3.14 × 10^6^, *P* = 0.23) or the presence of LPS (9.02 ± 3.4 × 10^6^ versus 7.92 ± 0.82 × 10^6^, *P* = 0.60).

In contrast, this increase in IFN-γ-secreting Treg was not accompanied by an increase in the proportion of Treg with capacity to secrete IL-17 (**Figure 3b**). Expanded populations of IFN-γ^+^ Treg after alloanergization and allorestimulation had high expression of the Th1 transcription factor T-bet and low expression of the Ikaros family transcription factor Helios, a phenotype which has been associated with high suppressive capacity in Type-1 inflammatory conditions^19^ (**Figure 3c**,**d**). As some recent studies have shown that IFN-γ^+^ FOXP3^+^ cells can lose suppressive function,^18^ we used γ-capture technology to purify populations of IFN-γ^+^ and IFN-γ^neg^ Treg (**Supplementary Figure S4**) for use in allosuppression assays. IFN-γ-secreting Treg retained potent allosuppressive capacity, demonstrating at least equivalence to IFN-γ^neg^ Treg on a per cell basis. Importantly, this suppressive function was retained in IFN-γ-secreting Treg expanded in proinflammatory conditions (**Figure 3e**).

Overall, these results suggest that proinflammatory conditions likely to be encountered *in vivo* do not significantly reduce the allosuppressive capacity of CD4^+^ Treg expanded after alloanergization. Furthermore the presence of LPS potentiates the expansion of a subpopulation of allosuppressive IFN-γ-secreting Treg which may facilitate the control of alloresponses in proinflammatory environments.

### Treg expanded after alloanergization possess differential migratory capacity

Having demonstrated that CD4^+^ Treg expanded after alloanergization maintain their allosuppressive function in proinflammatory environments, we next determined whether such cells had capacity to migrate to different tissues in order to suppress allogeneic T-cell responses effectively *in vivo*.

**Figure 4:**
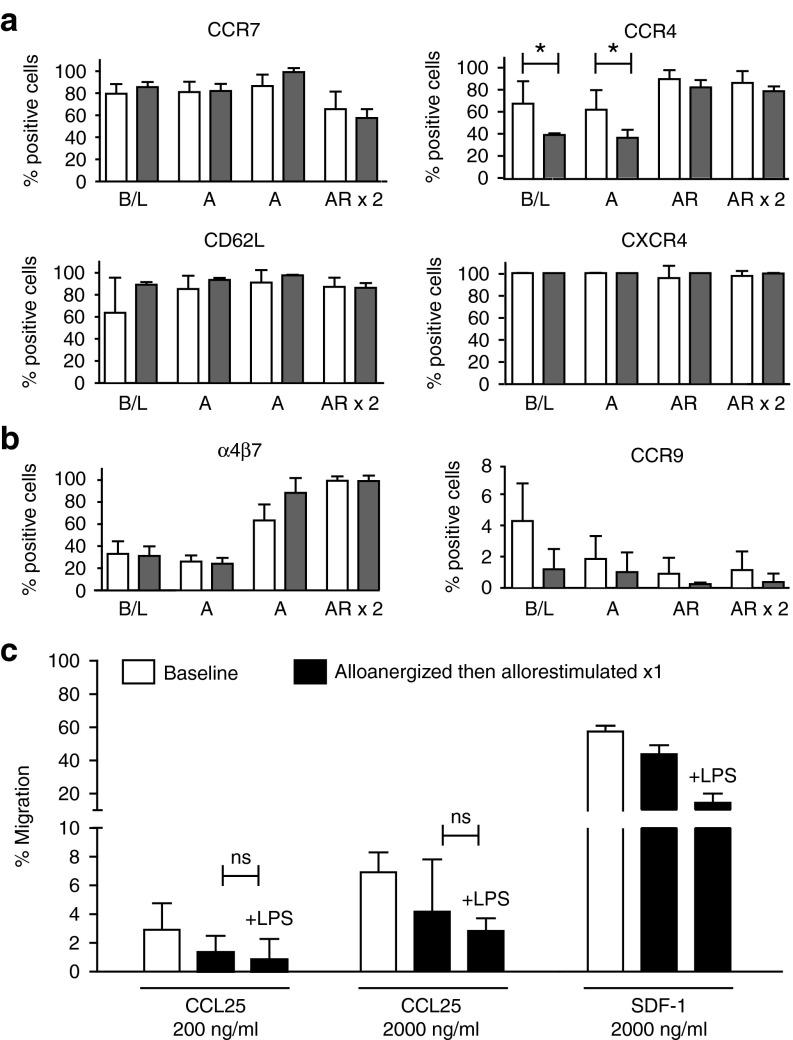
**Regulatory T-cells (Treg) expanded after alloanergization and allorestimulation display differential expression of molecules conferring organ-specific migratory capacity.** (**a**) Mean frequencies (±SD) of cells expressing CCR7, CD62L, CCR4 and CXCR4 expressed as a proportion of Treg at baseline (B/L) and after alloanergization (A) and subsequent allorestimulation (AR) in the absence (white bars) or presence (gray bars) of lipopolysaccharide (LPS) (*n* = 3–9). *P<0.05, (**b**) Mean frequencies (±SD) of cells expressing α_4_β_7_ and CCR9 expressed as a proportion of Treg at B/L and after A and subsequent AR in the absence (white bars) or presence (gray bars) of lipopolysaccharide (LPS) (*n* = 3–9). (**c**) CCL25 and SDF-1-specific chemotaxis of CD4^+^ Treg from baseline and after alloanergization and restimulation in the absence or presence of LPS. Bar charts depict mean (±SD) data from three different stimulator-responder pairs. ns, not significant.

First, we measured the expression of a range of chemokine receptor and adhesion molecules known to regulate the migration of Treg to anatomic sites of allogeneic T-cell priming and tissue damage.^20^ Significantly, expanded populations of CD4^+^ Treg maintained high levels of expression of both CD62L and C-C chemokine receptor type (CCR)7, molecules which facilitate migration to secondary lymphoid organ sites and the presence of LPS did not adversely impact on the level of expression of these molecules. Similarly, CXC-receptor 4 (CXCR4) and CCR4 which mediate Treg migration to the bone marrow and skin were expressed at high levels of expanded populations of Treg. Although LPS reduced the proportion of Treg expressing CCR4 in baseline and alloanergized Treg, LPS did not reduce expression of CCR4 in expanded populations of Tregs after alloanergization and allorestimulation and did not impact at all on levels of expression of CXCR4 (**Figure 4a**). These data are consistent with capacity of allosuppressive Treg expanded after alloanergization to migrate *in vivo* to sites of alloreactive effector T-cell priming and to two key sites of alloreactive T-cell-mediated tissue damage even in proinflammatory conditions.

Finally, we measured expression of molecules important to migration of Treg to the gastro-intestinal tract. The integrin α_4_β_7_, which facilitates migration of T-cells to gut-associated lymphoid tissue throughout the gastro-intestinal tract, was expressed on relatively low levels (20%) of Treg after alloanergization, but on larger proportions of Treg after allorestimulation (>80%), suggesting that this component of gut-trophism may be functional on expanded Treg. However, as expression of CCR9 plays an important additional and nonredundant role in migration of T-cells into the gut lamina propria, particularly in the small intestine,^21^ we also determined CCR9 expression on expanded populations of Treg before and after alloanergization. Significantly, CCR9 was expressed by <5% of baseline Treg and at even lower frequencies in expanded populations of Treg after alloanergization either in the absence or presence of LPS (**Figure 4b**).

In order to confirm that the low expression levels of CCR9 on Treg had functional impact, we performed chemotaxis assays using the CCR9 cognate ligand chemokine (C-C motif) ligand 25 (CCL25). Purified baseline Treg displayed low levels of migration toward CCL25 at a range of concentrations as compared to responsiveness to stromal cell-derived factor 1 (SDF-1) the cognate ligand for CXCR4. CCL25-specific chemotaxis of expanded Treg after alloanergization and allorestimulation remained low both in the despite maintenance of chemotaxis mediated by SDF-1/CXCR4 consistent with a selectively reduced ability for Treg to migrate to the lamina propria of the small intestine. LPS had no significant effect on levels of CCL25-specific migration in expanded populations of Treg (**Figure 4c**).

### Impaired CCR9-specific chemotaxis of allosuppressive Treg expanded after alloanergization could be partially corrected by pharmacological treatment

Finally, we sought to determine if the defective migratory phenotype of Treg expanded after alloanergization was tractable by pharmacological treatment. We chose a strategy of treatment with retinoic acid (RA) which upregulates the expression of CCR9 on T-cells.^22^ In addition, we examined the effect of the hypomethylating agent azacytidine (AZA) which increases RA receptor expression (and therefore might potentiate the effect of RA) in addition to stabilizing Treg function.^23,24,25^

**Figure 5:**
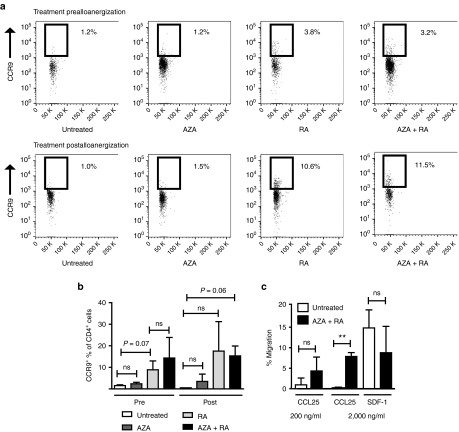
**Pharmacological treatment can partially correct defective CCR9-specific chemotaxis of regulatory T-cells (Treg) expanded after alloanergization.** (**a**) Azacitidine (AZA) and all-trans retinoic acid (RA) treatment prior to or after alloanergization increases the proportion of expanded Treg expressing CCR9. Illustrative dot pots are shown depicting CCR9 expression on CD4^+^ FOXP3^+^ Tregs after alloanergization with and without treatment with AZA, RA or both. Representative data are shown from one out of six experiments. (**b**) Mean frequencies (±SD) of cells expressing CCR9 expressed as a proportion of Treg after alloanergization without and with treatment with AZA, RA or both. ns, not significant. Individual *P* values where there was a trend to statistical significance (*P* > 0.05 < 0.10) are also shown. (**c**) CCL25 and stromal cell-derived factor 1 (SDF-1)-specific chemotaxis of CD4^+^ Treg from AZA/RA treated or untreated alloanergized PBMCs. Bar charts depict mean (±SD) data from three different stimulator-responder pairs. **P < 0.01, ns, not significant.

AZA treatment alone either before or after alloanergization had no significant effect on the proportion of CD4^+^ Treg expressing CCR9. RA treatment before or after alloanergization, increased the proportion of CD4^+^ Treg expressing CCR9. The largest increase was seen with RA treatment after alloanergization, although there was some variability of this effect. In contrast, combined RA and AZA treatment after alloanergization led to a more consistent increase in the frequency of the proportion of CD4^+^ Treg expressing CCR9 (from 1.53 ± 0.37% to 15.3 ± 4.5%, *P* = 0.06), **Figure 5a**,**b**). Importantly RA/AZA treatment also significantly improved Treg CCL25-specific chemotaxis without impairing CXCR4-specific chemotaxis (**Figure 5c**).

In addition, RA and RA/AZA treatment also increased the proportion of Treg expressing α_4_β_7_ which could serve to further improve gut-migratory capacity (**Supplementary Figure S5**).

## Discussion

We have examined critical functional aspects of a simple strategy to expand human alloantigen-specific Treg for therapeutic use after AHSCT.

Importantly, the presence of LPS did not reduce Treg expansion or function after alloanergization. It is therefore likely that Treg generated in this way retain capacity to suppress harmful effector T-cell-mediated immune responses in LPS-rich environments such as gastro-intestinal GvHD after AHSCT and autoimmune colitis. Although LPS has been shown to adversely affect FOXP3 protein stability by promoting ubiquitination through Stub1,^26^ our current findings are consistent with other studies showing that LPS can influence transcriptional and translational regulation of FOXP3 to enhance Treg proliferation and suppressive function.^27^ Our findings that IL-1β and IL-6 do not impede the generation or allosuppressive function of Treg after anergization also predict stability of function in inflamed gut tissue, as IL-1 signaling has recently been shown to trigger a developmental switch from induced Treg to proinflammatory Th17 cells in the gut microenvironment.^28^

Despite the retention of expansion and allosuppressive function, LPS-rich conditions did impact on the expanded populations of Treg after alloanergization by significantly increasing the proportion of Treg with capacity to secrete IFN-γ. Our findings that IFN-γ^+^ Treg expanded after alloanergization were potently allosuppressive are consistent with recent studies that have shown that human CD4^+^ Treg with capacity to secrete IFN-γ are critical for controlling T-cell responses in proinflammatory environments^19^ and are required for effective suppression of GvHD in murine transplant models.^17,29^ However, although subpopulations of IFN-γ^+^ Treg expanded after alloanergization may play an important role in controlling alloresponses in proinflammatory conditions, these cells represent only a small proportion of the expanded Treg pool. IFN-γ^neg^ Treg expanded after alloanergization were more abundant and possessed equivalent allosuppressive capacity to IFN-γ^+^ Treg. These findings highlight the heterogenous nature of expanded populations of allosuppressive Treg generated for therapeutic use in this way and how this dynamic pool can be impacted upon by the microenvironment.

An important aspect of the functional specialization of Treg is their ability to migrate to different organs. Animal models demonstrate that after AHSCT, donor Treg migrate to recipient secondary lymphoid organs where they suppress proliferation of alloreactive effector T-cells^30^ but Treg also directly infiltrate GvHD target tissues.^31,32^ Indeed, several studies have demonstrated that increased frequencies of chemokine receptor-positive Treg in the peripheral blood of patients inversely correlates with incidence and severity of organ-specific GvHD after AHSCT.^33^ Reassuringly, we found that Treg expanded after alloanergization retained high levels of expression of molecules key for migration to secondary lymphoid organs and target organs of GvHD including the skin and bone marrow and that levels of expression were not significantly altered in the presence of LPS.

Although Treg expanded after alloanergization had high expression of α_4_β_7_, important for migration to the gut, particulary the large intestine, the frequencies of Treg expressing CCR9, important in T-cell migration to the small intestine, were very low. Human studies of gut-specific migratory capacity of Treg after AHSCT have thus far been limited to expression of α_4_β_7_^33,34^ and no studies have directly examined the relationship between CCR9^+^ Treg and gut GvHD. However, there is evidence to suggest that low levels of CCR9 expression on Treg may be functionally significant in this setting, as increased levels of CCR9^+^ effector T-cells in patient peripheral blood post AHSCT have been associated with gut GvHD^35^ and CCR9 expression is essential for Treg-mediated control of inflammatory colitis in mice.^36^ A selective small-intestinal migratory defect is consistent with the preponderance of gastro-intestinal GvHD we observed in prior clinical trials of alloanergized donor T-cell therapy in the setting of AHSCT, despite detecting large *in vivo* expansions of allosuppressive Treg in patients' peripheral blood.^9^ Pretreatment with RA or AZA and RA partially corrected the low CCR9 expression levels, providing proof of principle that selective migratory defects that might limit the *in vivo* efficacy of antigen-specific Treg generated by anergization may be corrected by preinfusional pharmacological manipulation.

Despite providing valuable insight into the likely functional capacity of allosuppressive Treg generated after alloanergization, we must consider the limitations of our study. Our HLA-mismatched model is relevant to an increasing proportion of AHSCT procedures and to solid organ transplantation, but does not address minor histocompatibility- or self-antigen-specific Treg generated to selectively control T-cell responses in HLA-matched AHSCT or autoimmune disease. Furthermore, other studies have shown that CD8^+^ Treg are also important in controlling harmful alloresponses, particularly after solid organ transplantation.^37^ Our previous studies have shown that allosuppressive CD8^+^ Treg are also expanded after alloanergization^38^ but we have yet to investigate their functional stability and migratory capacity. Finally, we chose to limit our studies to *in vitro* models. Although migratory capacity of Treg can be more directly demonstrated in murine models, intrinsic differences between murine and human T-cells (most notably the differential expression of CD28^39^), and between allogeneic and xenogeneic T-cell responses^40^ limit the value of such model systems in this setting.

In conclusion, our study addresses two key potential mechanisms which might result in loss of *in vivo* efficacy of antigen-specific Treg generated by anergization; the stability of phenotype and function in the presence of relevant proinflammatory conditions, and differential capacity to migrate to tissues postinfusion. Our findings have obvious relevance for the application of this strategy to control harmful alloresponses after AHSCT, but also broad implications for wider application of this approach to generate antigen-specific Treg for use in other therapeutic settings.

## Materials and Methods

***Human cells.*** Donor peripheral blood mononuclear cells (PBMC) were isolated by density gradient centrifugation from pheresis filter collars. The study was approved by the London Research Ethical Committee (05/Q0605/140) and was conducted in accordance with the Declaration of Helsinki.

***Alloanergization of human PBMC.*** Responder PBMCs (10^7^) and equal numbers of 50Gy irradiated HLA-mismatched allogeneic PBMC were cocultured in the presence of 40 µg/ml second-generation CTLA4-Ig Belatacept (Nulojix, Bristol-Myers Squibb, New York, NY) at 10^6^/ml in Roswell Park Memorial Institute (RPMI) medium containing 10% AB human serum (GE Healthcare Life Sciences, Amersham Place, UK) in upright 25 cm^2^ flasks as described earlier.^9,41^ After 72 hours cultures were washed to remove costimulatory blockade and allorestimulated with either first party irradiated PBMC (original stimulators). Subsequent restimulations of alloanergized PBMCs were performed every 5 days. Where indicated LPS (1 µg/ml) (Sigma, Aldrich, St Louis, MO) or IL-1β (20 ng/ml) and IL-6 (30 ng/ml) (BioLegend, San Diego, CA) were added during the last 3 days of the restimulation culture period. In some experiments, responder cells were pretreated with 1 μmol/l azacitidine (a concentration that stabilized FOXP3 expression in Tregs without significantly impairing viability or proliferative capacity) and/or 1 μmol/l all-trans RA (both Sigma-Aldrich) for 3 days prior to or after the alloanergization process.

***Flow cytometry and cell sorting.*** Multicolor flow cytometry was employed using directly conjugated antibodies (**Supplementary Table S1**). CD4^+^ Treg were enumerated by coexpression patterns of CD25 and CD127 and by intranuclear FOXP3 as described earlier.^12^ For cytokine production cells were stimulated for 5 hours with stimulation cocktail and protein transport inhibitor cocktail (both from eBioscience, San Diego, CA). For background levels of cytokine secretion cells were stimulated with 1× Protein transport inhibitor cocktail only and values were subtracted from values for fully stimulated cells. Intracellular cytokine staining for cytokines and transcription factors was performed using the FOXP3 Fix/Perm Buffer staining kit from eBioscience.

Two-color flow cytometric cell sorting of Treg was performed with an ARIA II device from Becton Dickinson (Franklin Lakes, NJ). For methylation analysis Treg were defined as CD4^+^CD25^+^CD127^−^ or Tconventional, defined as CD4^+^CD25^−^CD127^+^, whereas for sorting of IFN-γ-secreting and nonsecreting populations of CD4^+^ Treg were defined based on CD25 and IFN-γ expression after prior immunomagnetic depletion of CD127^+^ cells to remove effector T-cells.

Dead cells were excluded using 4',6-diamidino-2-phenylindole for cell surface cytometry and Fixable Aqua dead cell stain (Life technologies, Paisley, UK) or Fixable V450-50 (eBioscience) dye for intracellular/intranuclear cytometry.

***Treg suppression assays.*** Immunomagnetically purified CD4^+^CD25^+^CD127^dim^ Treg (purity >80%, obtained using the CD4^+^CD25^+^CD127^dim/-^ Treg isolation kit II from Miltenyi Biotec (Bergisch Gladbach, Germany) from untreated, alloanergized and alloanergized allostimulated cultures were added to untreated responder PBMC responders in triplicate wells of 96-well-plates to which 50-Gy irradiated first party stimulators were added as described earlier.^12^ Proliferation was measured after 5 days by ^3^H-thymidine (Perkin Elmer, Waltham, MA) incorporation, with 1 µCi/well added during the last 18 hours. The percentage suppression of first party alloresponses was calculated as % suppression = 100 × (1−(cpm responders plus Treg/cpm responders alone)). Where indicated LPS (1 µg/ml), or IL-1β (20 ng/ml) and IL-6 (30 ng/ml) were added during the 5-day coculture period.

***Isolation of cytokine-secreting Treg.*** IFN-γ-secreting Treg were isolated using the cytokine secretion-cell enrichment and detection kit (Miltenyi Biotec) according to the manufacturer's instructions. Alloanergized allorestimulated PBMC were initially depleted of CD127-expressing effector T-cells by immunomagnetic separation using the CD4^+^CD25^+^CD127^dim/-^ Treg isolation kit II from MIltenyi Biotec. Cells were initially activated with 1× Cell stimulation cocktail (eBioscience) for 3 hours and then labeled with the cytokine catch reagent. During the secretion phase, cells were diluted in 10 ml culture medium/10^7^ cells to prevent capturing of cytokine from the neighboring cells. IFN-γ-labeled cells were then purified by flow cytometry. Suppression assays using cytokine-producing cells were performed as described earlier.

***Chemokine receptor expression and chemotaxis assays.*** Expression of chemokine receptors was assessed using flow cytometry with the antibodies listed in **Supplementary Table S1**. For chemotaxis assays culture medium containing CCL25 and SDF-1 (both Biolegend) was loaded into the bottom wells of 96-well ChemoTx Transwell polycarbonate microplates with 5 μm filter pores (Neuroprobe, Gaithersburg, MD). Purified CD4^+^CD25^+^CD127^−^ T-cells (3 × 10^4^) were loaded onto the upper wells of Transwell polycarbonate microplates and plates were incubated for 4 hours at 37 °C. To enumerate migrated cells, cells were counted at the bottom well and dead cells were excluded with trypan blue. A positive control for CCL25-specific chemotaxis is shown in **Supplementary Figure S6**.

***Statistical tests.*** Statistical analysis was performed using Prism version 5.0 software (GraphPad Software, La Jolla, CA). Two-tailed paired or unpaired *t*-tests were used to compare two groups. Equal variance was not assumed and Welch's correction used where appropriate. *P* values <0.05 were considered statistically significant. Error bars indicate SD.

**SUPPLEMENTARY MATERIAL** **Figure S1.** Expression of TLR4 and IL-1R1 on Treg. **Figure S2.** Expansion of CD25hiCD127lo CD4+ Treg after alloanergization is maintained in lipopolysaccharide (LPS)-rich conditions. **Figure S3.** Treg expanded after alloanergization remain allosuppressive in the presence of IL-1β and IL-6. **Figure S4.** Gating strategy for the identification and sorting of IFN-γ+ CD4+ Treg. **Figure S5.** Expression of α4β7 Treg expanded after alloanergization after treatment with azacytidine (AZA), retinoic acid (RA) or both. **Figure S6.** CCL25-specific chemotaxis control. **Table S1.** Flow cytometry antibodies.
