# Supplementary material for: Allospecific Tregs Expanded After Anergization Remain Suppressive in Inflammatory Conditions but Lack Expression of Gut-homing Molecules
Source: Mol Ther. 2016 May 3;24(6):1126–34. doi: 10.1038/mt.2016.64 (PMC4923329; doi:10.1038/mt.2016.64)
Supplement: Supplementary Figures and Table [file mt201664x1.pdf]

Supplementary Material

Figure S1

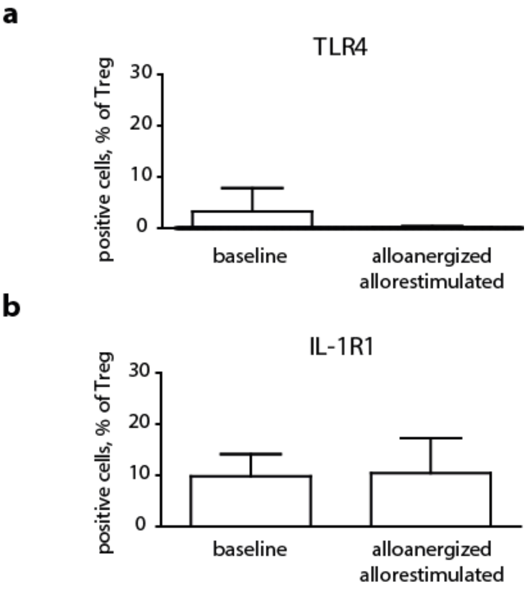

Figure S1 Expression of TLR4 and IL-1R1 on Treg. Mean (+/-sd) frequencies of TLR4 and IL-1R1$^+$ cells expressed as percentage of CD4$^+$ Treg at baseline, and after alloanergization and allorestimulation are shown. Results are shown for 3 HLA-mismatched stimulator- responder pairs.

Figure S2

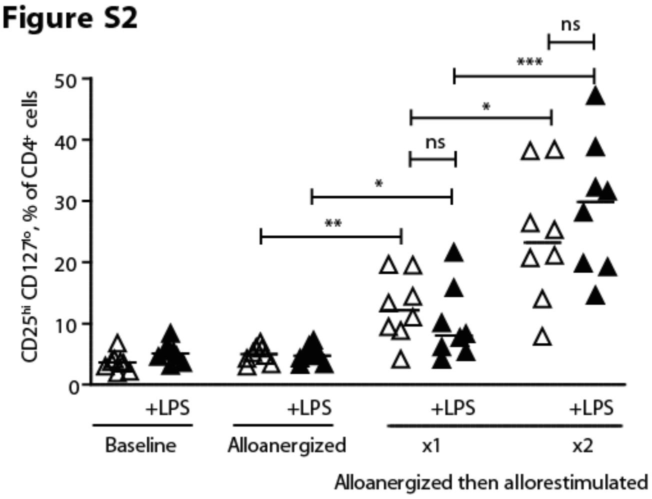

Figure S2: Expansion of CD25^{hi}CD127^{lo} CD4$^+$ Treg after alloanergization is maintained in lipopolysaccharide (LPS)-rich conditions

CD25^hi^CD127^lo^ Treg, expressed as percentage of total CD4$^+$ cells, after alloanergization and repeat exposure to alloantigen increase in the absence or presence of LPS. Graph depicts results from 8 different HLA-mismatched stimulator-responder pairs. *P* values are for two-tailed student's t test. Horizontal lines are medians. *, $p<0.05$, ** $p<0.01$, ns, not significant

Figure S3

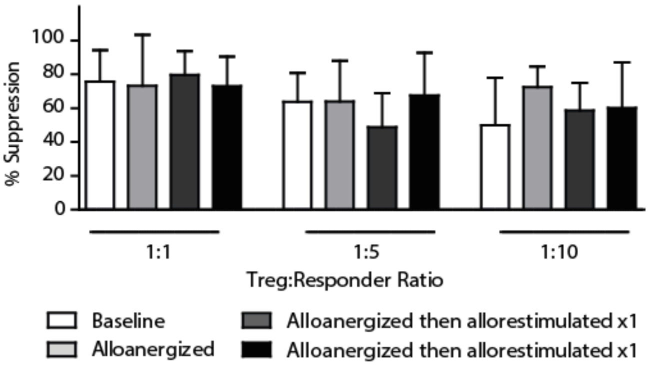

Figure S3: Treg expanded after alloanergization remain allosuppressive in the presence of IL-1β and IL-6

Allosuppressive capacity of CD4$^+$ Treg after alloanergization and subsequent allorestimulation of donor PBMCs in the presence of IL-1β and IL-6. Mean percentage suppression (± SD) of first-party alloproliferative responses of untreated autologous responder PBMCs by CD4$^+$ Treg from untreated, alloanergized and allorestimulated alloanergized PBMCs. Data are for 3-6 HLA-mismatched stimulator-responder pairs.

Figure S4

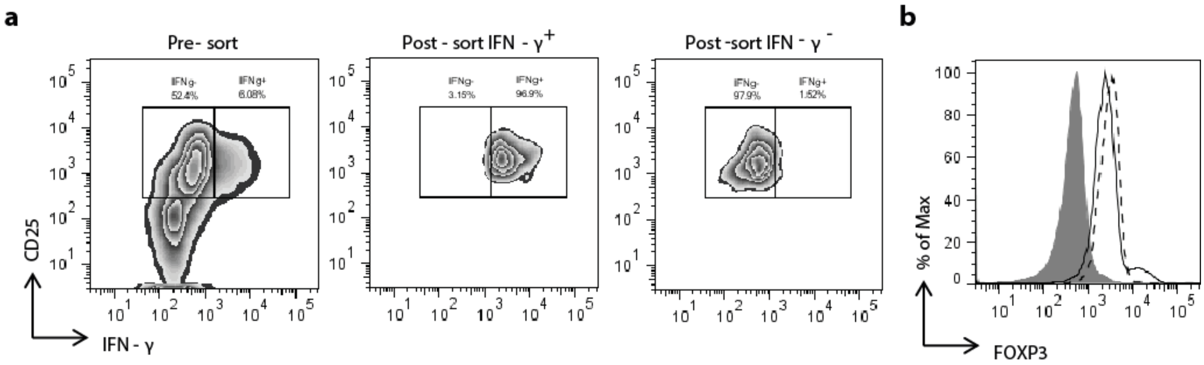

Figure S4: Gating strategy for the identification and sorting of IFN-γ+ CD4+ Treg

a). Bivariate dot plots showing CD25 and IFN-γ expression on CD4+CD127lo cells pre-sort (left panel) and post-FACS sort (middle and right panels) One representative example of four independent experiments is shown.

b) FOXP3 expression in the different subsets. Dashed unfilled histogram denotes CD25+ IFN-γ+ and solid unfilled histogram denotes CD25+ IFN-γneg cells. Filled histogram is CD25neg cells

Figure S5

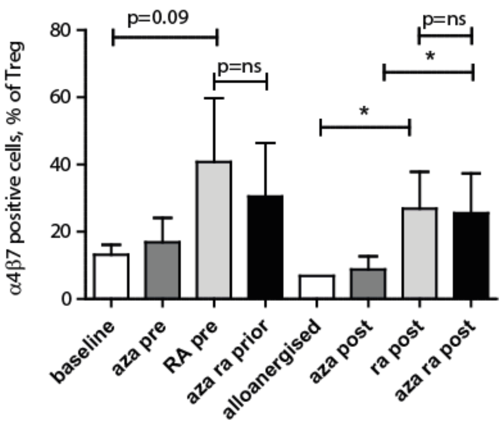

Figure S5 Expression of α4β7 Treg expanded after alloanergization after treatment with azacytidine (AZA), retinoic acid (RA) or both. Mean frequencies (±

SD) of cells expressing α4β7 expressed as a proportion of Treg after alloanergization without and with treatment with AZA, RA or both. * p<0.05, Ns, not significant Ns, not significant. Individual p values where there was a trend to statistical significance (p>0.05<0.10) are also shown.

Figure S6

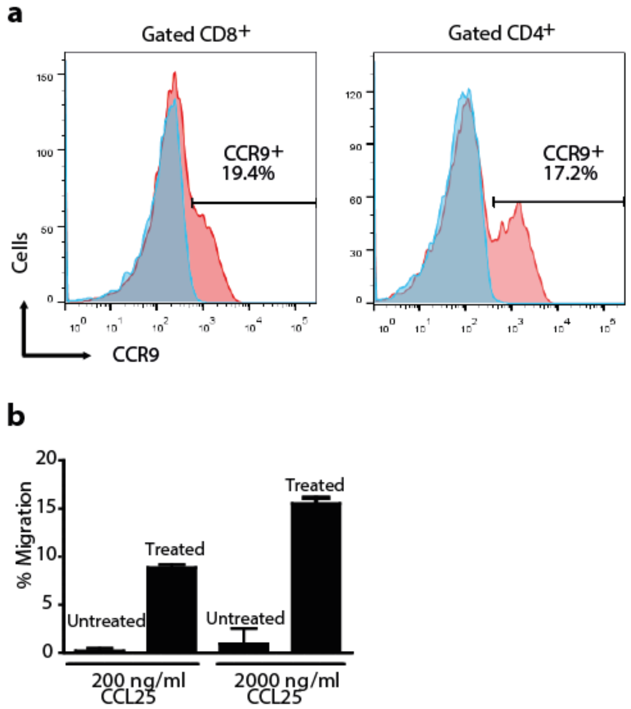

Figure S6: CCL25-specific chemotaxis control

A) Representative histograms showing expression of CCR9 on $CD4^+$ and $CD8^+$ T-cells subsets after activation of healthy donor PBMCs with CD3/CD28 beads and IL-2 in the presence (red histograms) or absence (blue histograms) of ATRA for 7 days). Representative data from one of three different donors.

B) CCL25 -specific chemotaxis of ATRA treated or untreated healthy donor PBMCs for 7 days in the presence of CD3/CD28 beads and IL-2. Bar charts combine data from 3 different stimulator-responder pairs. Mean percent chemotaxis (± SD) was calculated after subtraction of background migration.

Table S1: Flow cytometry antibodies

Table S1

Target	Fluorochrome	Clone	Manufacturer
**CD4**	FITC	M-T466	Miltenyi Biotec
**CD4**	ALEXAFLUOR700	RPA-T4	Biolegend
**CD4**	PE	M-T466	Miltenyi Biotec
**CD8**	APC	SK1	Biolegend
**CD25**	APC	4E3	Miltenyi Biotec
**CD39**	FITC	A1	Biolegend
**CD127**	PE	MB15-18C9	Miltenyi Biotec
**CD49d**	PE-Cy5	9F10	Biolegend
**CD127**	PACIFIC BLUE	A019D5	Biolegend
**CD161**	APC	HP-3G10	Biolegend
**CD62L**	FITC	DREG-56	Biolegend
**CCR4**	APC	L291H4	Biolegend
**CCR7**	APC	G043H7	Biolegend
**CCR9**	APC	L053E8	Biolegend
**CXCR4**	PE	12G5	Biolegend
**CTLA-4**	PE	12-1529	eBioscience

**FOXP3**	PE-Cy7	PCH101	eBioscience
**GARP**	PerCP-eFluor710	G14D9	eBIoscience
**GITR**	FITC	eBioAITR	eBioscience
**IFN-γ**	FITC	4S.B3	Biolegend
**IL-17**	PE	BL168	Biolegend
**Integrin β7**	PE	FIB504	Biolegend
**T-bet**	PerCP-Cy5.5	4B10	Biolegend
**TGF-β/LAP**	PerCP-Cy5.5	TW4-2F8	Biolegend
**Helios**	APC	22F6	Biolegend
**CD284 (TLR4)**	APC	HTA125	eBioscience
**IL-1R1**	ALEXAFLUOR700	Goat polyclonal	RnD Systems
